# Development of 15 microsatellite loci in the endangered *Streptanthus glandulosus* subsp. *niger* (Brassicaceae)

**DOI:** 10.1002/aps3.1215

**Published:** 2019-01-30

**Authors:** Sarah M. Swope, Alan E. Pepper, Geneva T. Lee, Brittany A. Burnett, Hannah M. Horten

**Affiliations:** ^1^ Department of Biology Mills College 5000 MacArthur Blvd. Oakland California 94613 USA; ^2^ Department of Biology Texas A&M University College Station Texas 77843 USA; ^3^ Department of Environmental Sciences Mills College 5000 MacArthur Blvd. Oakland California 94613 USA

**Keywords:** Brassicaceae, conservation, fragmentation, microsatellite, population genetics, *Streptanthus glandulosus* subsp. *niger*, *Streptanthus glandulosus* subsp. *secundus*, *Streptanthus tortuosus*

## Abstract

**Premise of the Study:**

The endangered *Streptanthus glandulosus* subsp. *niger* (Brassicaceae) is endemic to a single peninsula in California and threatened by fragmentation. We developed microsatellite markers to investigate genetic diversity in the two extant populations and the degree to which they have diverged from one another.

**Methods and Results:**

We used Illumina HiSeq high‐throughput sequencing to develop 15 microsatellite markers, 14 of which were polymorphic. These di‐ and trinucleotide repeats yielded one to 11 alleles per locus in 61 plants across the two populations. Levels of observed and expected heterozygosities ranged from 0.108 to 0.946 and 0.257 to 0.839, respectively. We demonstrated cross‐amplification in a second rare subspecies, *S. glandulosus* subsp. *secundus*, and in the widespread congener *S. tortuosus*.

**Conclusions:**

These are the first microsatellites reported for this subspecies, and they will aid in the inclusion of genetic information in conservation planning. Cross‐amplification was demonstrated in two related taxa, including one of conservation concern.

The genus *Streptanthus* Nutt. (Brassicaceae) is noteworthy for the remarkable morphological diversity, adaptations to unusual soil types, and rarity of its ~35 species (Cacho et al., 2014). *Streptanthus glandulosus* Hook. subsp. *niger* (Greene) Al‐Shehbaz, M. S. Mayer & D. W. Taylor is adapted to serpentine soils that are characterized by high concentrations of heavy metals and low concentrations of macronutrients. It has one of the most restricted ranges of all members of this group, confined to the 6‐km‐long Tiburon Peninsula (Marin County, California, USA). It is pollinated by bees and is capable of self‐pollination.

*Streptanthus glandulosus* subsp. *niger* occupied a large portion of the Tiburon Peninsula prior to suburban development in the mid‐20th century but now persists as two populations separated by 1.5 km of dense housing and is listed as Endangered at both the state and federal levels (California Department of Fish and Wildlife, 2014; U.S. Fish and Wildlife Service, 2019). The population at Middle Ridge Park is smaller than the population at Old St. Hilary's Preserve. The total number of plants ranges from ~500 to 3000 depending on annual precipitation (Swope, unpublished data). Nothing is known about the genetic diversity or structure of the two remaining populations or gene flow between them. Although microsatellites are widely used to answer these questions and may be useful in conservation planning, none exist for this subspecies.

## METHODS AND RESULTS

Fresh leaves were collected from 40 individuals at Old St. Hilary's Preserve and 24 at Middle Ridge Park, stored in separate envelopes at room temperature with silica beads (PolyLam Products, Williamsville, New York, USA), and extracted within one week of collection. DNA was extracted by placing 3–4 mm of dried leaf tissue in 300 μL of a 10% Chelex solution (Bio‐Rad Laboratories, Hercules, California, USA), vortexing samples for 10 s, then spinning them for another 10 s to ensure that plant material was in the solution. The solution was incubated at 65°C for 10 min, followed by another round of vortexing for 10 s. Finally, we centrifuged the samples for 10 s to separate contaminants and Chelex beads from the DNA in the supernatant. We diluted the supernatant from the Chelex extraction 1 : 1 with distilled H_2_O.

A microsatellite library was created using extracted genomic DNA from three individuals, one from the Middle Ridge Park population and two collected from opposite ends of the larger Old St. Hilary's Preserve population. Due to restrictions on collecting, our specimens were vouchered with existing herbarium collections (Appendix 1). DNA was sent to the QB3 Genomics Sequencing Laboratory at the University of California, Berkeley, for shotgun library preparation and 2 × 300‐bp paired‐end Illumina HiSeq genome sequencing. Approximately nine million FASTQ reads per sample were filtered for minimum length (50 bp) and trimmed for quality using Trimmomatic version 0.23.3 (Bolger et al., 2014) using a window size of 4 bp with a threshold average quality of Phred = 25, and a minimum score of 15 at the leading and trailing ends of the read. Reads were assembled de novo with ABySS (Simpson et al., 2009) using a *K*‐mer size of 41 bp, a minimum bubble identity of 0.9, and a minimum contig size of 300 bp. To avoid acquisition bias, we selected loci that were identified as microsatellites in all three genomic DNA assemblies using misa.pl (Beier et al., 2017) in a search for dimeric and trimeric repeats with a minimum of six repeat units. Loci were pre‐screened for polymorphism by “round‐robin” mapping trimmed reads from each genomic DNA to the other two genome assemblies using Bowtie2 (Langmead and Salzberg, 2012) using pre‐set default parameters, followed by variant calling using NGSEP version 3.0.3 (Perea et al., 2016) with a ploidy of two. Evidence from transcriptome sequencing indicates that a common ancestor to *S. glandulosus* subsp. *niger* and other members of the tribe Thelypodieae underwent a major (possibly whole genome) duplication event ~8–10 mya (Kagale et al., 2014; Hawkins et al., 2017). A diploid chromosome number of 2*n* = 28 is almost universally conserved across the tribe (Warwick and Al‐Shehbaz, 2006). Rare reports of unusual karyotypes (e.g., 2*n* = 14, 2*n* = 56) show no phylogenetic pattern (Cacho et al., 2014) and thus appear to be sporadic in nature. In *Caulanthus amplexicaulis* S. Watson, a 2*n* = 28 sister taxon to *S. glandulosus* subsp. *niger*, 250 of 258 microsatellite markers (97%) produced a single amplification product from each of two distinct homozygous inbred lines. Considered together, these lines of evidence justify the selection of diploid as a ploidy parameter. Genomic coordinates identified as microsatellites by misa.pl were thus filtered for the presence of dimeric and trimeric repeats in all three assemblies that were in all cases called by NGSEP as “STR” and “INDEL” with a minimum confidence score of 100. Forty‐one microsatellite loci met these criteria.

Primers for the 41 loci were designed to have 35–55% GC content, a target melting temperature of 64–65°C (salt‐adjusted, 50 mM NaCl), and an amplicon size of 90–150 bp. A subset of 23 loci were screened for amplification by electrophoresing the products on a 4% SFR agarose gel (VWR Life Science, Philadelphia, Pennsylvania, USA) with an Invitrogen 100‐bp ladder (Invitrogen, Carlsbad, California, USA) using nine *S. glandulosus* subsp. *niger* individuals. Of those 23 loci, 15 produced consistent and robust amplification products and thus were suitable for genotyping. Sequence library data were deposited into the National Center for Biotechnology Information Sequence Read Archive (BioProject ID PRJNA503999).

PCR conditions were optimized using a Bio‐Rad T100 Thermal Cycler (Bio‐Rad Laboratories). Amplification reactions were singleplexed in a final volume of 25.5 μL containing approximately 2 ng of DNA, 12.5 μL of Q5 High‐Fidelity DNA Polymerase (New England BioLabs, Ipswich, Massachusetts, USA), 1 μL of Milli‐Q water (MilliporeSigma, Burlington, Massachusetts, USA), and 0.5 μM of each forward and reverse primer. The PCR program consisted of one cycle of denaturation at 98°C for 30 s; followed by 34 cycles at 98°C for 40 s, 60°C for 30 s, 72°C for 20 s; and an extension phase at 72°C for 1 min. Forward primers were fluorescently labeled at the 5′ end with HEX or FAM (Eurofins, Louisville, Kentucky, USA). PCR products were diluted (1 : 30 to 1 : 75) and run with 0.2 μL of GeneScan 600 LIZ‐labeled internal size standard (Thermo‐Fisher Scientific, Waltham, Massachusetts, USA) and 9 μL of Hi‐Di Formamide (Applied Biosystems, Foster City, California, USA) in a single lane and analyzed using an Applied Biosystems 3730XL DNA Analyzer. Allele sizes (Table 1) and peaks were determined using Peak Scanner 2.0 (Thermo‐Fisher Scientific).

**Table 1:** Characteristics of 15 microsatellite loci developed for *Streptanthus glandulosus* subsp. *niger*

Locus	Primer sequences (5′–3′)	Repeat motif	Allele size range (bp)	*T*_a_ (°C)	Fluorescent label	GenBank accession no.
Sn50	F: CTATGTCCTCCATAAACCTCAACGC	(CAA)_7_	132–138	68	FAM	MG029359
	R: GTTGAAGGTAAGGTACATTTGCCCTG					
Sn255	F: GGTTCCAGAACAGAAGAACTCAGC	(CT)_13_	138–182	66	FAM	MG029360
	R: GCATTATTATCTGCTCTCTCATGTTGG					
Sn262	F: CATCTAACTTCTCTGTTAGATGAAAACG	(CT)_9_	147–175	64	FAM	MG029357
	R: CTCATACTTCTCTGCTTGAAGTTGG					
Sn313	F: GATGTCCACACAAACCATATGTACC	(AT)_10_	157–171	65	HEX	MH316554
	R: CAAACTAAATAGCTGAAAAGGGTTAAAAGG					
Sn347	F: CAATTGATTCCTTGATCTTCAATATTACC	(TA)_9_	95–105	63	FAM	MG029355
	R: GATCGCAGAAGTTACCGATGAAGG					
Sn430	F: GGCAGAGTGCATTAGGAGTTGACG	(TA)_8_	131–145	67	FAM	MG029364
	R: CTAAACGGTAAAACCCAACATATGGTG					
Sn463	F: AAGAGAGGTTATCATGGCTAATGAAGC	(AAC)_4_	82–109	67	FAM	MG029358
	R: CGACCGACGAGAAACCGAACG					
Sn558	F: GGAGACATGCAGCATCTCAACG	(TC)_11_	92–114	64	HEX	MG029356
	R: TCAACCCAAATTTAGAAAATCTATTGATTTGG					
Sn588	F: GAAATCGTTGCCACTACCTTCTGC	(TCC)_8_	128–182	63	HEX	MG029361
	R: GAGAAGGAATACGAAGTAGAGAAC					
Sn715	F: CCCGTCAATCTCTAACGTCTAGC	(TA)_9_	92–118	68	FAM	MH316555
	R: TAGATGATTCAACCCACTGATTGTGC					
Sn803	F: GTTTAATATGGTTCAAAGCAGGACTGG	(TA)_8_	141–197	63	FAM	MG029362
	R: CTTTATTGGCTAATTGAATATTTGTAATCTCC					
Sn1015	F: TCATCGTATAACGGGAAAGGATCCA	(TA)_4_	96–154	66	HEX	MH316558
	R: CACGAAATTCGTGGTAGTATTTCGAG					
Sn1434	F: TGTATCTGTATTATCCTCACATGTAAATCC	(TA)_7_	81	64	HEX	MH316552
	R: CTGGAAGATTGTGACCTTATTCTTGG					
Sn1618	F: GAAATAGAGAGAGGAATCTCTGTCTG	(CTT)_6_	139–169	65	FAM	MG029363
	R: GTGGGTTAGCACTACAATCTGGTG					
Sn2378	F: CAACAGTGTATATCAATTTGATATCACTGG	(TAA)_7_	124–220	64	FAM	MH316557
	R: CGTGCTTAGACCTACGAAGTTCC					

Note

*T*_a_ = annealing temperature.

We used GenAlEx (Peakall and Smouse, 2012) to measure the number of alleles per locus and observed and expected heterozygosities. We used GENEPOP 4.7.0 (Rousset, 2008) to test for linkage disequilibrium and deviations from Hardy–Weinberg equilibrium using a sequential Bonferroni correction for multiple tests, and MICRO‐CHECKER 2.2.3 (van Oosterhout et al., 2004) to assess the presence of null alleles.

The average number of alleles across all loci was 5.70 (±0.46 SE; range 1–11) (Table 2). Three loci (Sn313, Sn715, and Sn803) may harbor null alleles. We detected significant linkage disequilibrium between loci Sn558 and Sn463. Of the 15 loci, 12 deviated from Hardy–Weinberg equilibrium at the Old St. Hilary's Preserve site and five deviated at the Middle Ridge Park site (Table 2).

**Table 2:** Genetic diversity of 15 microsatellites developed for *Streptanthus glandulosus* subsp. *niger* and tested for cross‐amplification in *S. glandulosus* subsp. *secundus* and *S. tortuosus*.^a^

Locus	*Streptanthus glandulosus*								Cross‐amplification^c^	
	Old St. Hilary's Preserve (*n* = 40)				Middle Ridge Park (*n* = 24)					
	*A*	*H*_o_ b	*H*_e_	*N*	*A*	*H*_o_ b	*H*_e_	*N*	*S. glandulosus* subsp. *secundus* (*n* = 6)	*S. tortuosus* (*n* = 10)
Sn50	3	0.405	0.465	37	2	0.391	0.440	23	180–220	180–200
Sn255	7	0.378	0.330	37	9	0.583	0.485	24	150–180	160–210
Sn262	5	0.265**	0.687	34	7	0.391**	0.504	23	143–151	147–153
Sn313	6	0.450**	0.729	30	3	0.300	0.485	20	—	—
Sn347	6	0.500**	0.593	36	5	0.292	0.444	24	100	80–140
Sn430	8	0.946**	0.666	37	6	0.833**	0.793	24	136–142	134–144
Sn463	4	0.378*	0.515	37	7	0.667	0.717	24	88–94	100–140
Sn558	7	0.361**	0.766	36	7	0.500	0.715	24	86–94	150–300
Sn588	4	0.541**	0.717	37	5	0.500	0.556	24	128–144	182
Sn715	7	0.667**	0.757	30	10	0.714**	0.788	20	+	—
Sn803	10	0.548**	0.733	31	11	0.800**	0.839	20	—	—
Sn1015	5	0.243**	0.669	37	6	0.375**	0.679	24	—	—
Sn1434	1	0.000^NA^	0.000	37	1	0.000^NA^	0.000	24	81	81
Sn1618	6	0.297**	0.291	37	3	0.417	0.487	24	142–154	169
Sn2378	6	0.108**	0.463	37	4	0.238	0.257	21	180–220	200–220

Note

— = no amplification; + = amplification of more than two amplification products; *A* = number of alleles; *H*_e_ = expected heterozygosity; *H*_o_ = observed heterozygosity; *n* = number of individuals sampled; *N* = number of successfully genotyped individuals.

a Locality and voucher information are provided in Appendix 1.

b Significant deviation from Hardy–Weinberg equilibrium (*P < 0.05, **P < 0.01, NA = not applicable).

c Values represent allele size range in base pairs.

We tested all 15 loci for amplification in another rare subspecies (*S. glandulosus* subsp. *secundus* Greene) and a congener (*S. tortuosus* Kellogg) using the same protocol. *Streptanthus glandulosus* subsp. *secundus* is restricted to approximately three dozen populations in two neighboring counties, whereas *S. tortuosus* spans nearly the entire 1200‐km length of California. Eleven loci successfully amplified in both taxa (Table 2), producing one or two resolvable amplification products per individual, suggesting they amplified a single locus. Three loci produced no amplification products in either species, and one locus amplified more than one product in *S. glandulosus* subsp. *secundus* (Table 2).

## CONCLUSIONS

We developed microsatellite markers for the endangered *S. glandulosus* subsp. *niger* and two congeners. Optimization of these markers will be useful in quantifying the genetic diversity in the two remaining populations of *S. glandulosus* subsp. *niger* and the degree to which these populations have diverged from one another. These markers may also be useful in comparative studies with *S. glandulosus* subsp. *secundus*, which is also of conservation concern.

## AUTHOR CONTRIBUTIONS

S.M.S. conceived of the project, collected leaf tissue, supervised the lab and field work, conducted allele scoring and analyses, and wrote the manuscript. A.E.P. conducted the whole‐genome shotgun sequencing, contig assembly, and variant calling, and co‐authored the manuscript. G.T.L., B.A.B., and H.M.H. conducted the lab work and reviewed the manuscript.

## Data Availability

Sequence library data were deposited in the National Center for Biotechnology Information (NCBI) Short Read Archive (BioProject ID PRJNA503999). Sequence information for the developed primers has been deposited to NCBI; GenBank accession numbers are provided in Table 1.

## APPENDIX 1 Voucher and location information for species used in the development and evaluation of microsatellite markers for *Streptanthus glandulosus* subsp. *niger*.^a^

**Table:**

Taxon	Collection locality	Geographic coordinates^b^	Voucher specimen accession no.^c^
*Streptanthus glandulosus* Hook. subsp. *niger* (Greene) Al‐Shehbaz, M. S. Mayer & D. W. Taylor	Tiburon Peninsula, Marin Co., CA, USA	37°52′N, 122°27′W	CAS‐BOT‐BC87245, CAS‐BOT‐BC87246, CAS‐BOT‐BC87253, UC163931, JEPS2879, JEPS73882, JEPS77161, JEPS9284
*Streptanthus glandulosus* subsp. *secundus* Greene	Carson Ridge, Marin Co., CA, USA	37°57′N, 122°37′W	CAS‐BOT‐BC87281, UC1492744
	Bolinas Road, Marin Co., CA, USA	37°57′N, 122°37′W	CAS‐BOT‐BC87272
	Terra Linda‐Sleepy Hollow, Marin Co., CA, USA	38°01′N, 122°34′W	CAS‐BOT‐BC87286
*Streptanthus tortuosus* Kellogg	Gold Lake region, Plumas Co., CA, USA	39°56′36″N, 121°08′03″W	CAS‐BOT‐BC87795
	Confluence of Rock Creek and north fork of Feather River, Plumas Co., CA, USA	39°54′01″N, 121°21′31″W	CAS‐BOT‐BC87797
	Confluence of Granite Creek and north fork of Feather River, Plumas Co., CA, USA	39°57′20″N, 121°17′44″W	CAS‐BOT‐BC87783
	East fork of Feather River, north of Red Hill, Plumas Co., CA, USA	40°03′21″N, 121°12′28″W	CAS‐BOT‐BC87785

Due to the protected status of *S. glandulosus* subsp. *niger*, new voucher specimens were not collected for this study; the vouchered specimens listed here are representative of the plants used in this study.

a Geographic coordinates for *S. glandulosus* subsp. *niger* and *S. glandulosus* subsp. *secundus* have been reduced to minutes due to the protected status of these taxa.

b Vouchers with the prefix CAS are deposited at the California Academy of Sciences Herbarium (CAS), San Francisco, California, USA; vouchers with the prefixes UC and JEPS are deposited at University and Jepson Herbaria (JEPS), University of California, Berkeley, California, USA.

## References

[aps31215-bib-0001] Beier, S. , T. Thiel , T. Münch , U. Scholz , and M. Mascher . 2017 MISA‐web: A web server for microsatellite prediction. Bioinformatics 33: 2583–2585. 28398459 10.1093/bioinformatics/btx198 PMC5870701

[aps31215-bib-0002] Bolger, A. M. , M. Lohse , and B. Usadel . 2014 Trimmomatic: A flexible trimmer for Illumina sequence data. Bioinformatics 30: 2114–2120. 24695404 10.1093/bioinformatics/btu170 PMC4103590

[aps31215-bib-0003] Cacho, N. I. , A. M. Burrell , A. E. Pepper , and S. Y. Strauss . 2014 Novel nuclear markers inform the systematics and the evolution of serpentine use in *Streptanthus* and allies (Thelypodieae, Brassicaceae). Molecular Phylogenetics and Evolution 72: 71–81. 24333439 10.1016/j.ympev.2013.11.018

[aps31215-bib-0004] California Department of Fish and Wildlife . 2014 California Threatened and Endangered plant profiles: Tiburon jewelflower (Streptanthus glandulosus ssp. niger). Website https://www.wildlife.ca.gov/Conservation/Plants/Endangered/Streptanthus-glandulosus-ssp-niger [accessed 8 January 2019].

[aps31215-bib-0005] Hawkins, A. K. , E. R. Garza , V. A. Dietz , O. J. Hernandez , W. D. Hawkins , A. M. Burrell , and A. E. Pepper . 2017 Transcriptome signatures of selection, drift, introgression, and gene duplication in the evolution of an extremophile endemic plant. Genome Biology and Evolution 9: 3478–3494. 29220486 10.1093/gbe/evx259 PMC5751042

[aps31215-bib-0006] Kagale, S. , S. J. Robinson , J. Nixon , R. Xiao , T. Huebert , J. Condie , D. Kessler , et al. 2014 Polyploid evolution of the Brassicaceae during the Cenozoic era. Plant Cell 26: 2777–2791. 25035408 10.1105/tpc.114.126391 PMC4145113

[aps31215-bib-0007] Langmead, B. , and S. L. Salzberg . 2012 Fast gapped‐read alignment with Bowtie 2. Nature Methods 9: 357–359. 22388286 10.1038/nmeth.1923 PMC3322381

[aps31215-bib-0008] Peakall, R. O. D. , and P. E. Smouse . 2012 GenAlEx 6.5: Genetic analysis in Excel. Population genetic software for teaching and research—an update. Bioinformatics 28: 2537–2539. 22820204 10.1093/bioinformatics/bts460 PMC3463245

[aps31215-bib-0009] Perea, C. , J. F. De La Hoz , D. F. Cruz , J. D. Lobaton , P. Izquierdo , J. C. Quintero , B. Raatz , and J. Duitama . 2016 Bioinformatic analysis of genotype by sequencing (GBS) data with NGSEP. BMC Genomics 17(Supplement 5): 498. 27585926 10.1186/s12864-016-2827-7 PMC5009557

[aps31215-bib-0010] Rousset, F. 2008 GENEPOP: A complete re‐implementation of the GENEPOP software for Windows and Linux. Molecular Ecology Resources 8: 103–106. 21585727 10.1111/j.1471-8286.2007.01931.x

[aps31215-bib-0011] Simpson, J. T. , K. Wong , S. D. Jackman , J. E. Schein , S. J. Jones , and I. Birol . 2009 ABySS: A parallel assembler for short read sequence data. Genome Research 19: 1117–1123. 19251739 10.1101/gr.089532.108 PMC2694472

[aps31215-bib-0012] U.S. Fish and Wildlife Service . 2019 Endangered species database. Website https://www.fws.gov/endangered/species/us-species.html [accessed 8 January 2019].

[aps31215-bib-0013] van Oosterhout, C. , W. F. Hutchison , D. P. M. Wills , and P. Shipley . 2004 MICRO‐CHECKER: Software for identifying and correcting genotyping errors in microsatellite data. Molecular Ecology Notes 4: 535–538.

[aps31215-bib-0014] Warwick, S. I. , and I. A. Al‐Shehbaz . 2006 Brassicaceae: Chromosome number index and database on CD‐Rom. Plant Systematics and Evolution 259: 237–248.

